# Transcriptomic response of *Anopheles gambiae* sensu stricto mosquito larvae to Curry tree (*Murraya koenigii*) phytochemicals

**DOI:** 10.1186/s13071-020-04505-4

**Published:** 2021-01-02

**Authors:** Clarence M. Mang’era, Fathiya M. Khamis, Erick O. Awuoche, Ahmed Hassanali, Fidelis Levi Odhiambo Ombura, Paul O. Mireji

**Affiliations:** 1 Department of Biochemistry and Molecular Biology, Egerton University, Njoro Campus, PO Box 536-20115, Egerton, Kenya; 2 Department of Biochemistry, Microbiology and Biotechnology, School of Pure and Applied Sciences, Kenyatta University, Ruiru Campus, PO Box 43844-00100, Nairobi, Kenya; 3 International Centre of Insect Physiology and Ecology (ICIPE), Duduville Campus, Kasarani, PO Box 30772-00100, Nairobi, Kenya; 4 Department of Biological Sciences, Meru University of Science and Technology, PO Box 972-60200, Meru, Kenya; 5 Department of Chemistry, School of Pure and Applied Sciences, Kenyatta University, Ruiru Campus, PO Box 43844-00100, Nairobi, Kenya; 6 Biotechnology Research Institute-Kenya Agricultural and Livestock Research Organization, PO Box 362-00902, Kikuyu, Kenya

**Keywords:** *Anopheles gambiae* s.s, Growth disruption, Mosquito larvae, Differential gene expression, *Murraya koenigii*

## Abstract

**Background:**

Insect growth regulators (IGRs) can control insect vector populations by disrupting growth and development in juvenile stages of the vectors. We previously identified and described the curry tree (*Murraya koenigii* (L.) Spreng) phytochemical leaf extract composition (neplanocin A, 3-(1-naphthyl)-l-alanine, lumiflavine, terezine C, agelaspongin and murrayazolinol), which disrupted growth and development in *Anopheles gambiae *sensu stricto mosquito larvae by inducing morphogenetic abnormalities, reducing locomotion and delaying pupation in the mosquito. Here, we attempted to establish the transcriptional process in the larvae that underpins these phenotypes in the mosquito.

**Methods:**

We first exposed third-fourth instar larvae of the mosquito to the leaf extract and consequently the inherent phytochemicals (and corresponding non-exposed controls) in two independent biological replicates. We collected the larvae for our experiments sampled 24 h before peak pupation, which was 7 and 18 days post-exposure for controls and exposed larvae, respectively. The differences in duration to peak pupation were due to extract-induced growth delay in the larvae. The two study groups (exposed *vs* control) were consequently not age-matched. We then sequentially (i) isolated RNA (whole larvae) from each replicate treatment, (ii) sequenced the RNA on Illumina HiSeq platform, (iii) performed differential bioinformatics analyses between libraries (exposed *vs* control) and (iv) independently validated the transcriptome expression profiles through RT-qPCR.

**Results:**

Our analyses revealed significant induction of transcripts predominantly associated with hard cuticular proteins, juvenile hormone esterases, immunity and detoxification in the larvae samples exposed to the extract relative to the non-exposed control samples. Our analysis also revealed alteration of pathways functionally associated with putrescine metabolism and structural constituents of the cuticle in the extract-exposed larvae relative to the non-exposed control, putatively linked to the exoskeleton and immune response in the larvae. The extract-exposed larvae also appeared to have suppressed pathways functionally associated with molting, cell division and growth in the larvae. However, given the age mismatch between the extract-exposed and non-exposed larvae, we can attribute the modulation of innate immune, detoxification, cuticular and associated transcripts and pathways we observed to effects of age differences among the larvae samples (exposed *vs* control) and to exposures of the larvae to the extract.

**Conclusions:**

The exposure treatment appears to disrupt cuticular development, immune response and oxidative stress pathways in *Anopheles gambiae* s.s larvae. These pathways can potentially be targeted in development of more efficacious curry tree phytochemical-based IGRs against *An. gambiae* s.s mosquito larvae.
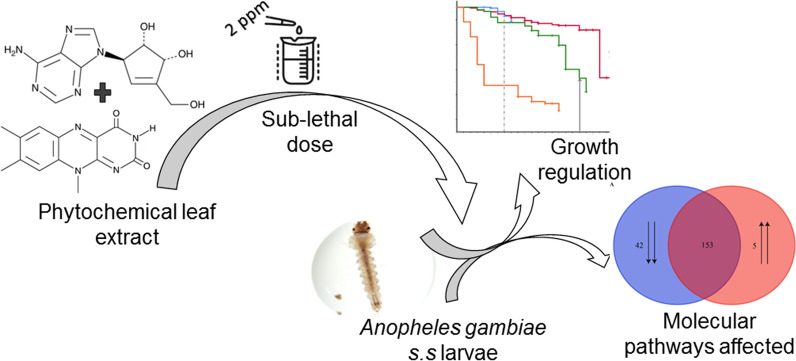

## Background

*Anopheles gambiae* s.s is a vector of human malaria disease responsible for > 400,000 deaths worldwide, mostly (> 93%) in sub-Sahara Africa [1]. Most control strategies against *An. gambiae* target the adult stage of the vector [2] and rely on pyrethroids, organochlorides, organophosphates and carbamates among other insecticides and against which the vector has developed resistance [3]. The resistance has adversely affected integrated mosquito vector control strategies [4] and necessitated a search for alternative control agents, including phytochemicals, that target immature stages of the vector [5]. This approach has largely been unexplored [6, 7]. Targeting the immature stages can perturb mosquito population dynamics, consequently contributing to reduced vectorial capacity and local malaria transmission [8]. However, this approach requires environmentally friendly agents because of the aquatic nature of mosquito larval habitats typically shared with many non-target organisms that include humans, livestock and crops. The phytochemicals provide better candidates for new classes of insecticides because they (i) consist of variable components with diverse mechanisms of action that diminish the odds of development of resistance in the mosquito to the phytochemicals [9], (ii) generally have minimal acute toxicity to vertebrates and (iii) are environmentally safe [10]. Current effective phytochemical agents targeting developmental stages in *An. gambiae* include larvae and/or pupae growth inhibitors [11, 12], larvicides [13] and ovicides [14]. These phytochemicals were derived from various mahogany (*Meliaceae*) plant families [15, 16], Japanese cedar (*Cryptomeria japonica*) [17], black pepper (*Piper nigrum*) [18], Indian wild pepper (*Vitex trifolia*) [19] and wild honeysuckle tree [20]. We recently assessed the effects of exposure of larval stages of *An. gambiae *sensu stricto to sub-lethal doses of leaf extracts from the curry tree (*Murraya koenigii*) and described the phytochemical composition of the extract [11]. We established that the exposure induced gross morphogenic abnormalities in the larvae and emergent adults, reduced larval locomotion and delayed pupation in the mosquito. We further established that the leaf extract was a blend of at least neplanocin A, 3-(1-naphthyl)-l-alanine, lumiflavine, terezine C, agelaspongin and murrayazolinol alkaloid phytochemicals [11].

We, therefore, undertook the present study to establish the molecular process that putatively underpins the phenotypes above that were observed in the *An. gambiae* s.s larvae following the exposure to the sub-lethal doses of phytochemical leaf extracts from the curry tree. However, the effect of delayed pupation on larvae by the extract [11] suggested a differential rate of growth between extract-exposed and non-exposed larvae, presenting unique challenges in age-matching larvae from the two populations. Consequently, larvae of chronologically similar age are potentially physiologically different (at different larval instar developmental stages) because of the differential growth rates between the populations. Second, such toxicity assessments are typically performed on L_3_/L_4_ instar larvae (irrespective of numerical age of the larvae) since growth inhibition compounds have the most profound effect on insects at that metamorphosis stage of their growth [21]. Faced with this challenge, we selected 24 h preceeding peak pupation (L_3_/L_4_ instar larvae) in both populations as the appropriate sampling point that were potentially physiologically similar but of dissimilar age. The consequence of this approach is that our findings reflect responses to an inevitable combination of age differences between the larval populations and the xenobiotic (extract) challenge. We identified potential molecular target candidates that can aid in research and development of more efficacious IGRs against *An. gambiae* s.s mosquito larvae.

## Methods

### Preparation of *An. gambiae* s.s larvae biological samples for RNA isolation

We obtained *An. gambiae *s.s mosquito larvae from a mosquito colony maintained at the International Centre of Insect Physiology and Ecology (ICIPE), Nairobi, Kenya. This mosquito colony was originally initiated, reared and maintained at Mbita Point Research and Training Centre of ICIPE, Homa Bay County, Kenya, from adults collected in 1996 at Njagi village in south-east Tanzania [22]. The colony was transferred and used to establish the *An. gambiae *s.s colony we used for our study at ICIPE, Nairobi, Kenya, in December 2000. This colony had never been previously exposed to any insecticide or growth-regulating xenobiotics, including plant extracts, and was thus considered susceptible to known insecticides and other xenobiotics. We followed standard procedures for rearing *Anopheles* mosquitoes [23]. We reared all life stages in an insectary (28 ± 2 °C, 75–80% relative humidity) at the Animal Rearing and Quarantine Unit of ICIPE, Nairobi, Kenya. From the day of emergence, we provided adult mosquitoes with a 10% sugar solution soaked in cotton wool. We fed 3-day-old adult female mosquitoes on bovine blood using an artificial membrane feeding method [24]. Approximately 2–3 days later, we placed oviposition dishes in the cage containing gravid females. We placed the eggs on water and surrounded them with floating wax paper to prevent them from getting stranded on the sides of the hatching tray. We typically placed about 30 mg pulverized Tetramin fish food (Tetra GmbH, Melle, Germany) per pan of water twice daily (three times daily after reaching the third larval stage). We collected the pupae daily, transferred them to bowls with water and placed the bowls in cages for adult emergence. For our study, we maintained the larvae on 0.3 mg powdered Tetramin fish meal per larvae per day as recommended for *An. gambiae *s.s larvae [25] under the insectary conditions. This quantity of food was sufficient to sustain the larvae for 24 h while establishing minimal residual food that could potentially interfere with bioavailability of the extract to the larvae.

We exposed the larvae to curry tree leaf extract following the protocol of Mang’era et al. [11] with relevant modifications for our current applications. The extract consisted of a natural blend of alkaloid phytochemicals (neplanocin A, 3-(1-naphthyl)-l-alanine, lumiflavine, terezine C, agelaspongin and murrayazolinol) and induced morphogenetic abnormalities, reduced locomotion and delayed pupation (8-day delay) in the larvae [11]. Briefly, we initiated this process by establishing the temporal range of toxicity of specific phytochemical extract concentration (2 ppm) to late third-early fourth instar larvae (L_3_/L_4_) of the mosquito. The temporal range was critical to our determination of optimal duration of exposure of the larvae to putatively elicit molecular responses to our extract at the L_4_ instar larvae just before pupation. We selected this concentration (2 ppm) since we have previously identified it as sub-lethal to the L_3_/L_4_ of the mosquito in an extract toxicity range (median lethal concentration) assessment [11]. For this purpose (establishing the temporal range of toxicity), we first solubilized the extract in absolute ethanol and diluted the resultant solution to 2 ppm in a total volume of 100 ml solution of distilled water (in a 250-ml glass beaker). Our overall ethanol concentration was 0.2% (v/v) absolute ethanol in the distilled water, based on the quantity of absolute ethanol we initially used to solubilize the extract. We then placed 20 L_3_/L_4_ instar larvae of the mosquito into the solution, consequently exposing them to the extract and serving as our exposed treatment. We similarly placed 20 L_3_/L_4_ instar larvae in 100 ml of distilled water with a similar concentration of absolute ethanol in a water beaker (in a 250-ml glass beaker) that served as our control treatment. The absolute ethanol concentration equivalence between the treatments potentially assisted in contrasting the effect of the extract from that of the solubilizing agent in downstream analyses. We prepared five replicates each of the treatment and control and monitored daily mortality and pupation of these larvae in both treatments under insectary conditions.

**Fig 1:**
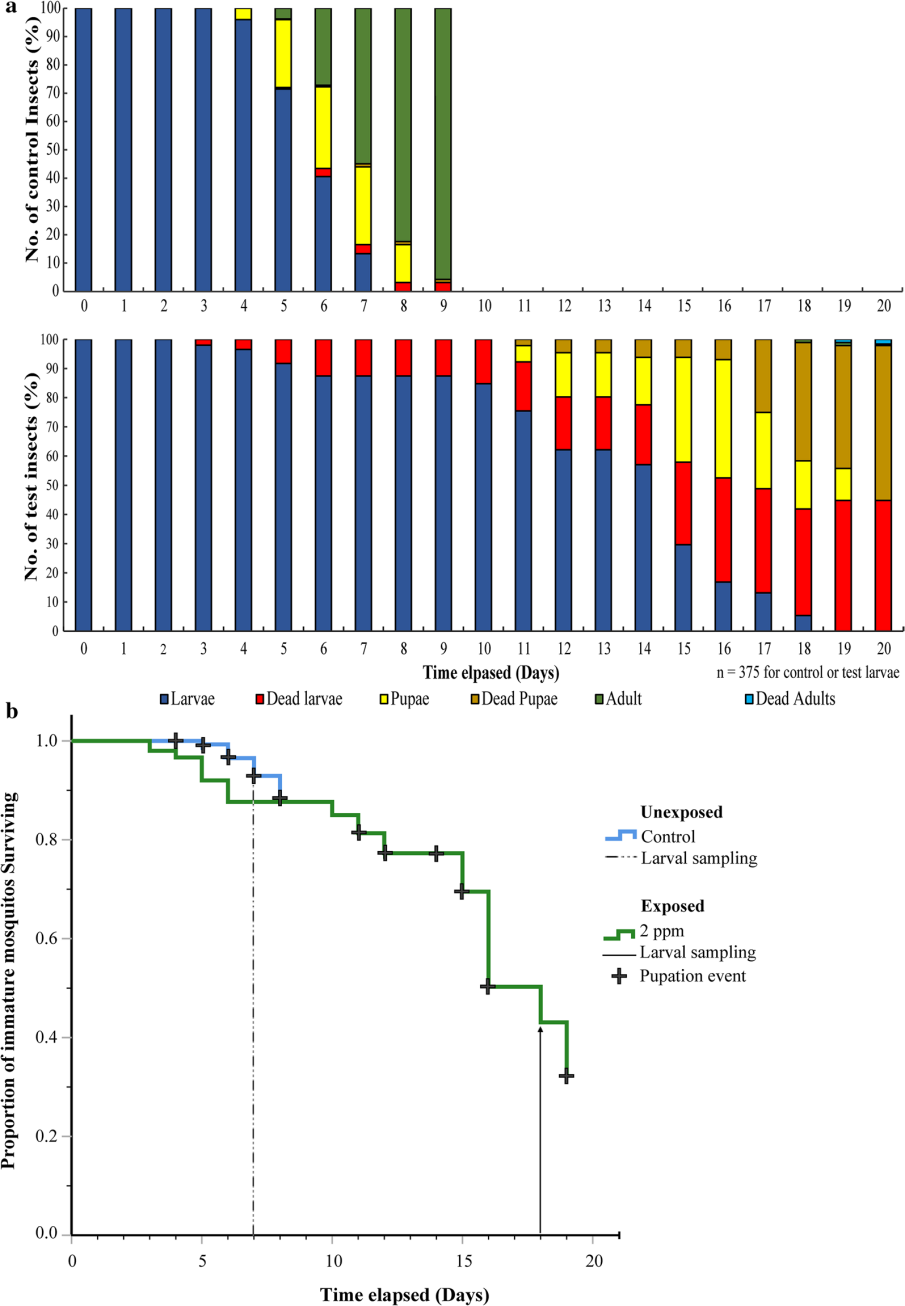
**a** Bar graph showing daily pupation/mortality ratios for unexposed and exposed L_3_/L_4_ instar larvae throughout the experiment. **b** Kaplan-Meier plot showing survival trends of unexposed and exposed (2 ppm) larvae

Our findings revealed peak pupation of the larvae 7 and 18 days post-exposure to our control and exposed treatments, respectively (Fig. 1a, b), suggesting that (i) the extract appeared to delay growth in the larvae and, consequently, the exposed and associated control larvae were potentially physiologically (L_3_/L_4_ instar larvae) similar 7 and 18 days post-exposure (dpe), respectively. At this point 81 and 53% of the larvae had pupated or eclosed (Fig. 1a) for control and exposed larvae populations, respectively, indicating that the remaining respective proportions would potentially be appropriate L_4_ instar larvae for further analyses in the definitive phase of our studies. The significant extension of larval phase (delayed pupation) by the extract exposure presented us with unique challenges in selection of the sampling points for the RNA extraction and subsequent RNA-Seq experiments. We reckoned that age-matching by sampling the larvae from either population 7 days post-exposure (peak pupation in control population) would constitute comparison between L_2_ exposed and L_3/4_ control larvae populations; hence, the outcome/results would be confounded by differences in the physiological states between the populations. The alternative was to consider exposed and control larvae populations 7 and 18 dpe, respectively, as physiologically equivalent (both at L_3_/L_4_ instar larvae developmental stage). The results from this approach would be confounded by the age differences between the larvae populations. Balancing these options and their consequences, we selected the latter option since growth inhibition compounds typically have the most profound effect on insects at the L_3_/L_4_ instar larval metamorphosis stage (physiologically matched) of their growth [26]. Consequently, our molecular results would reflect responses to the inevitable combination of age differences between the larvae populations and the xenobiotic (extract) challenge. The exposed will hereafter refer to this larvae population (extract-exposed but not age-matched larvae population).

For assessment of molecular events associated with the exposure to the phytochemical extract, we repeated similar treatments (exposed and control), but in 15 independent biological replicates of 25 larvae per treatment, to enhance the odds of obtaining sufficient surviving larvae for the subsequent molecular studies, given potential 55% mortality and 59% pupation as revealed by our preliminary studies (Fig. 1a) and the need to minimize crowding of the larvae in the beakers. We monitored and recorded larval survivorship at 24-h intervals, indicating numbers of larvae alive, dead or moribund, and removed the dead larvae. Since we were interested in two biological replicates each for the control or exposed treatments for our subsequent RNA-Seq molecular comparative analyses, we randomly and separately assigned five replicates from each treatment (exposed or control) into two groups of five treatments each. We performed these assignments 7 or 18 dpe for the control or exposed treatments, respectively. From the 50 and 49 L_4_ instar larvae survivors from control and exposed treatments, respectively (Fig. 1b), we pooled surviving larvae in each group into two separate 1.5-ml reaction tubes, constituting our two biological replicates of surviving larvae for subsequent RNA isolations and analyses. The replicates consisted of 25, 25, 25 and 24 larvae in each of control replicates 1 and 2 and exposed replicates 1 and 2, respectively. We quickly centrifuged the larvae in the tubes at 15,300 rcf for 1 min (Eppendorf AG 5417R centrifuge, Hamburg, Germany) to pellet the larvae and facilitate removal of the residual water. We then snap froze the larval pellet in liquid nitrogen until RNA isolation.

### Isolation and sequencing of *An. gambiae *s.s RNA

We isolated total *An. gambiae *s.s RNA from the two biological independent replicates (from control or exposed larvae) by mechanically crushing the larvae using disposable RNAse-free plastic pestles in ISOLATE II RNA Mini Kit buffer (Bioline, Merdian Life Sciences, London, UK) following the manufacturer’s protocol. We then treated resultant total RNA with TURBO DNase™ (Ambion Life Technologies, TX, USA), following the manufacturer’s instructions, to remove potentially contaminating DNA that could confound our subsequent RNA-Seq analysis. We verified the quality and integrity of the RNA samples using Agilent Bioanalyzer 2100 (Agilent, Palo Alto, CA, USA) according to the manufacturer’s instructions. We sourced services for cDNA library preparation from the total RNA and subsequent sequencing of the libraries from Macrogen, Korea (Geumcheon-gu, Seoul, Republic of Korea). Therein, cDNA libraries were prepared from 900 ng (per replicate) of high-quality total RNA (RNA integrity number between 8 and 9.7) using the Illumina TruSeq Stranded mRNA LT sample preparation kit (Illumina, Hayward, CA, USA) according to the manufacturer’s instructions. The cDNA libraries were then sequenced (101-bp paired-end read) on an Illumina HiSeq 2500 sequencer (Illumina, Hayward, CA, USA) according to the manufacturer’s instructions. Low-quality reads (< 100 base pairs) and adapter sequences in the libraries were then removed using Illumina build software (Illumina, Hayward, CA, USA). Overall, four transcriptome sequences were generated from our samples (two each for control or exposed larvae). We deposited the raw transcriptomes at the Sequence Read Archive (SRA) of the National Center for Biotechnology Information (NCBI), USA, under study accession number PRJNA560504.

### Identification and validation of responsive transcripts in *An. gambiae *s.s RNA to *M. koenigii* leaf extract

We separately assessed the quality of each library using FastQC software [27] and used the results to filter and trim out low-quality sections of the reads using CLC Genomic Workbench version 9.0 software (CLC Bio, Aarhus, Denmark). We then obtained the protein-coding gene set AgamP4.4 of *An. gambiae *s.s from VectorBase [28] and mapped the filtered and trimmed reads to the gene set (AgamP4.4) using RNA-Seq analysis procedures in CLC Genomic Workbench software version 9.0 (CLC Bio, Aarhus, Denmark) as described previously [29]. Briefly, we mapped the reads through settings that allowed two mismatches per read (with a maximum of 10 hits per read), with at least 80% of each read matching the gene at 95% identity. We then used reads per kilobase per million (RPKM) mapped reads as a proxy for quantity (abundance) of transcripts [30] for (1) assessment of baseline transcriptional processes that underpin the larval developmental stage in the mosquito (in the absence of xenobiotics) and (2) comparison of relative expression of the genes between libraries from control and exposed larvae. We established the relative number of reads for each transcript in relation to the total read counts for each RNA-seq library to calculate the *p*-value based on Baggerly's test method following Bonferroni analysis [31]. We then determined the relative fold change (FC) of transcripts between the control and exposed mosquito larvae as a ratio of the RPKM values and normalized based on the number of reads obtained from each library using an inbuilt algorithm in CLC Genomic Workbench. The normalized values were used in this study. We considered transcripts differentially expressed (DE) between the libraries if they had a normalized value of at least (i) 1.5-fold change, (ii) corrected *p* < 0.05 false discovery rate (FDR), (iii) ten RPKM and (iv) a support of ten unique read mappings. We defined fold change as a ratio of RPKM values between those from exposed and control larvae libraries. We thus considered and categorized these transcripts as specific to control or exposed larval libraries.

We also conducted gene ontology (GO) enrichment analyses of differentially expressed transcripts in exposed relative to control larvae libraries using gProfiler [32] to establish pathways, networks and interactions associated with transcripts induced or suppressed by the exposure of larvae to the extract. For these analyses, we set our thresholds to a significance of *p* = 0.05 (to retrieve all GO terms under the biological process, molecular function and cellular component) and ordered query to identify specific functional terms associated with the most significant changes for our query [33]. Since the extract predominantly induced morphogenetic abnormalities in the larvae [11] that we putatively associate a priori with cuticular metabolism [34, 35], which changes with developmental stages in insects [36], we further focused our analyses on cuticular proteins (CPs). We consequently detected and classified putative structural CPs among the DE transcripts through blastp [37] searches against the AgamP4.4 gene set of *An. gambiae *s.s from VectorBase [28] and CutProtFam-Pred CPs family prediction tool [38, 39]. The tool classified the CPs into protein families that (i) exhibited Rebers and Riddiford (R&R) Consensus sequences (CPR) [38, 40], (ii) were based on a conserved region with a 44 amino acid motif (CPF) and (iii) were analogous to peritrophins with chitin-binding domains (CPAP). The tool further classified the CPR into RR-1, RR-2 and RR-3 subfamilies based on variations in their consensus amino acid sequences in the R&R domain [38] and identified cuticular proteins of low complexity (CPLC), which encompass CPLCA, CPLCP and CPLCX among our differentially expressed transcripts.

### Validation of transcriptome expression profiles using RT-qPCR

#### Reverse transcriptions of larval RNA transcripts

We further evaluated whether our RNA-Seq analysis results could be independently replicated using the quantitative reverse transcription polymerase chain reaction (RT-qPCR) technique as an independent tool. We randomly selected DE larval transcripts in our RNA-seq library and compared their relative fold changes to those we obtained for the same transcripts through RT-qPCR. Briefly, we generated five independent biological replicates (exposed and control) of the larvae, extracted and cleaned the respective total RNA libraries from our control and exposed larvae *ab initio*. We used the same methods and procedures we used to prepare the biological samples for the RNA-seq component of our study for sample preparations and total RNA extraction. We then reverse transcribed 1 μg of the total RNA using the iScript™ cDNA synthesis kit (BIO-RAD, Hercules, USA) on the Arktik thermal cycler (Thermo Scientific, USA), according to the manufacturer’s protocol.

#### Selection of candidate transcripts for RT-qPCR

We randomly selected eight DE transcripts for validation of differential expression. These transcripts were significantly induced or suppressed in the exposed larvae library relative to the control library to encompass and validate differential expression in both directions (up- and downregulated genes) of the libraries. We also selected four transcripts that were neutral (neither induced nor suppressed in the exposed library relative to the control library) as potential internal reference neutral/loading controls [41]. These reference transcripts consisted of CLIP-domain serine protease, glyceraldehyde 3-phosphate dehydrogenase (*gapdh*) and two uncharacterized genes (Additional file 1: Table S1) from VectorBase [28]. We ensured that all these transcripts were abundantly expressed in the RNA-seq libraries (based on their RPKM values) to ensure that their expression levels would be within the sensitivity of our Stratagene MX3005P RT-qPCR machine (Agilent Technologies, CA, USA). We obtained DNA sequences of respective genes from VectorBase [28] using the respective gene IDs (Additional file 1: Table S1) and designed primers (Additional file 1: Table S1) from these sequences *in silico* using primer3 software [42]. In all cases, we ensured that the melting (*T*_m_) and annealing temperatures of the respective forward and reverse primers generated were similar, as determined by pDRAW32 version 1.1.142 software (http://www.acaclone.com) (Additional file 1: Table S1).

#### Real-time qRT-PCR of genes for DE transcripts

We first interrogated reference transcripts for their stable expression by performing RT-qPCR in three technical replicates for each of the five biological replicates on our Strategene MX3005P RT-qPCR machine (Agilent Technologies, CA, USA) using Fast SYBR Green I Master Mix (Applied Biosystems, Carlsbad, CA) according to the manufacturer's instructions. We performed the PCR in reaction volumes of 10 μl for each replicate consisting of 1 μg cDNA template in three independent replicates with 5 μl of Fast SYBR Green Master Mix (Applied Biosystems, Carlsbad, CA, USA) in the presence of 0.4 picomoles of specific primers for the respective candidate reference transcripts. We carried out the reactions in a RT-qPCR thermal cycler (Stratagene MX3005P, Agilent Technologies, CA, USA) according to the manufacturer’s instructions. We involved thermo-cycling conditions that included an initial step of 95 °C for 10 min, 40 cycles of 95 °C for 30 s, 55.0–63.2 °C (Additional file 1: Table S1) for 45 s and 72 °C for 1 min, followed by one cycle of 95 °C for 1 min, 55 °C for 30 s and 95 °C for 30 s for all the genes. We then assessed stability (non-differential expression) of these reference transcripts using BestKeeper software [43]. From this assessment, we identified *gapdh* and CLIP-domain serine protease transcripts as less variable [with a standard deviation of crossing point (CP) of 0.56 and 0.65, respectively] among the reference transcript candidates. We thus adopted these two genes as our internal housekeeping transcripts for assessment of expression of the eight randomly selected transcripts. We then separately performed the RT-qPCR for each of these transcripts under similar reaction and thermocycling conditions as had been previously employed in the assessment for stable expression of the reference transcripts above, but with *gapdh* and CLIP-domain serine protease as internal reference/loading controls.

#### Data analysis

We computed and compared relative expression (means, fold changes and *p*-value) of the eight transcripts using Relative Expression Software Tool (REST)-384 version 2 software [44]. We then used these data to evaluate validity of the transcriptomes by comparing the fold changes obtained here (RT-qPCR) to those obtained earlier (RNA-Seq) for the eight genes through Pearson correlation analysis.

## Results

### Survivorship of *An. gambiae* mosquito larvae exposed to the extract and non-exposed control

We have summarized and presented data on the survivorship of *An. gambiae* mosquito larvae exposed to the extract and non-exposed control in Fig. 1a, b. We observed initial pupation at 4 and 11 dpe for control and exposed larvae, respectively. Most pupae (96%) from the extract-exposed larvae populations did not eclose. At the point of sample collection, 53 and 81% of the larvae had pupated or eclosed for the extract-exposed and control populations respectively. Therefore, at peak pupation for the exposed population (18 dpe) the larvae were potentially abnormal. Thus, the subsequent RNA-Seq libraries consisted of the normal control and the abnormal exposed larvae populations.

### Mapping statistics of larval RNA-seq reads on the *An. gambiae *s.s gene set

**Fig 2:**
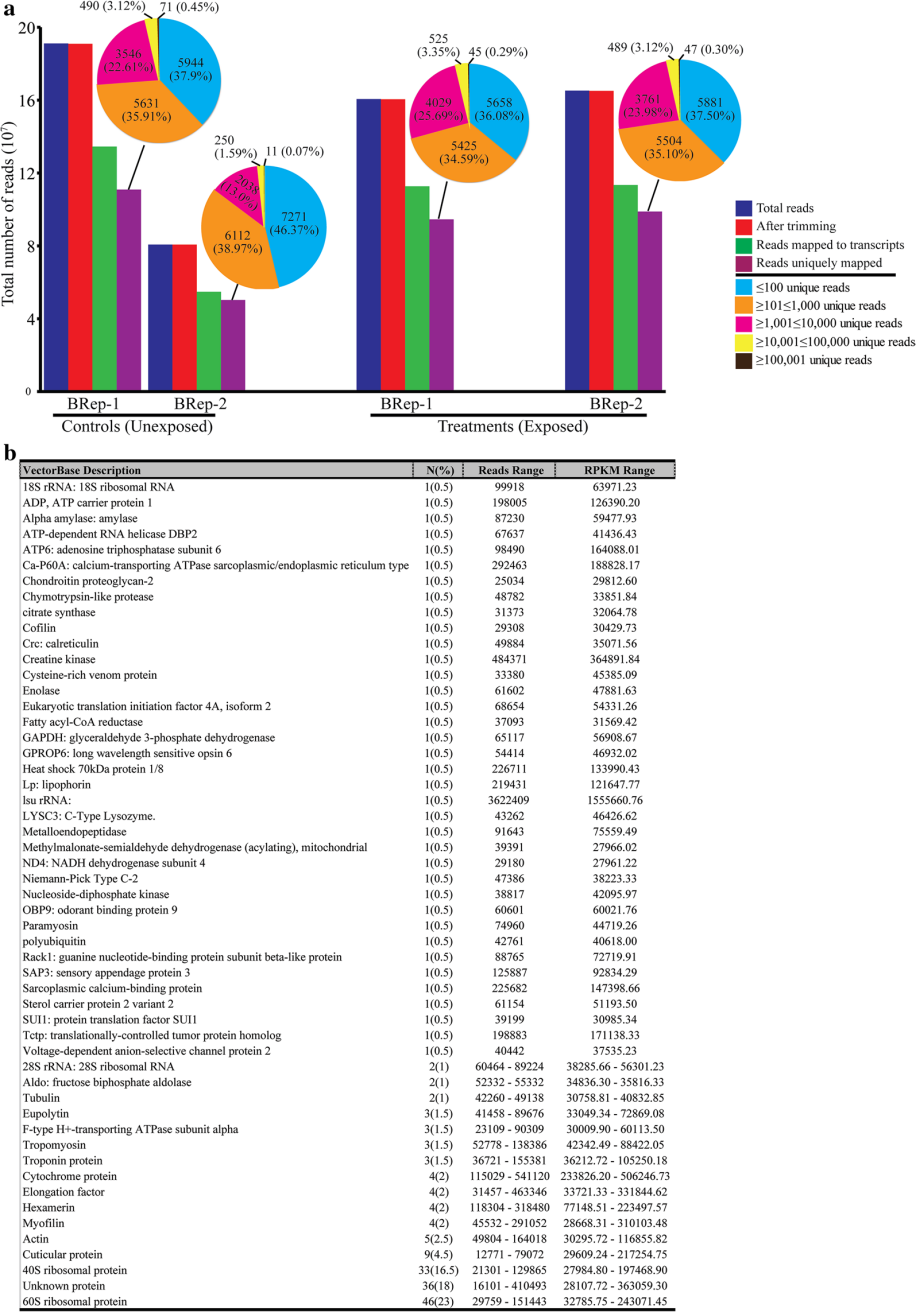
Overview of RNA-seq analysis of *Anopheles gambiae* larvae exposed to the *Murraya koenigii* bioactive fraction. **a** Processing of RNA-seq reads from *Anopheles gambiae* larvae and mapping statistics of the reads. **b** Summary of the top 200 most abundantly expressed (RPKM) genes in the control larvae

We achieved a Pearson correlation coefficient of 0.978 (Additional file 3: Text S1), indicating a 97.8% correlation in expression between RNA-seq and qRT-PCR results and effectively validating the transcriptomes. We obtained ≈ 80 to 191 million reads from sequencing the *An. gambiae *s.s larval libraries (Fig. 2a). The variation in the number of reads obtained is due to the different depths we achieved in the sequencing of each library. We successfully mapped 65–70% of these reads onto the protein-coding gene set AgamP4.4 of *An. gambiae *s.s from VectorBase [28], among which 58.1–62.3 % mapped uniquely to specific transcripts. Most of the transcripts had between 101 and 10,000 uniquely mapped reads (Fig. 2a).

**Fig 3:**
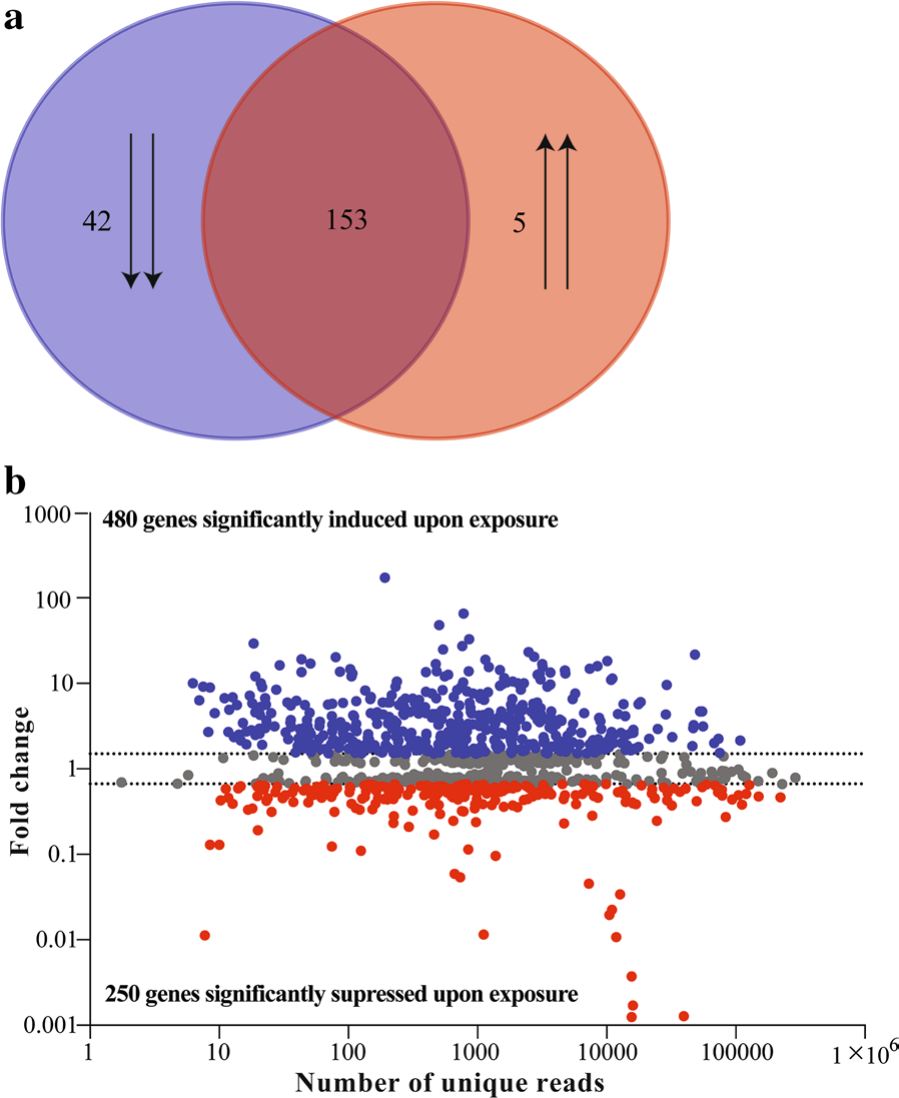
Differential expression of transcripts exhibiting significant expression in exposed larvae. **a** Spatial distribution of differential expression in the top 200 most abundant (RPKM) genes of the transcriptome. **b** The MA plot showing differentially expressed genes between control and exposed *An. gambiae* larvae

Our assessment of baseline transcriptional processes that underpin the larval developmental stage in the mosquito (in the absence of xenobiotics) identified ribosomal, cuticular, hexamerins, cytochrome, elongation factor and muscle-related proteins as abundantly expressed transcripts (Fig. 2b; Additional file 2: Table S2). About 23.5 % (47) of the 200 top-most abundantly expressed transcripts were differentially expressed between the libraries, among which expression of most (42) of the transcripts were suppressed in the exposed larvae libraries relative to those of control larvae (Fig. 3a).

### Differentially expressed and enriched pathways between control and exposed *An. gambiae* larvae libraries

**Fig. 4:**
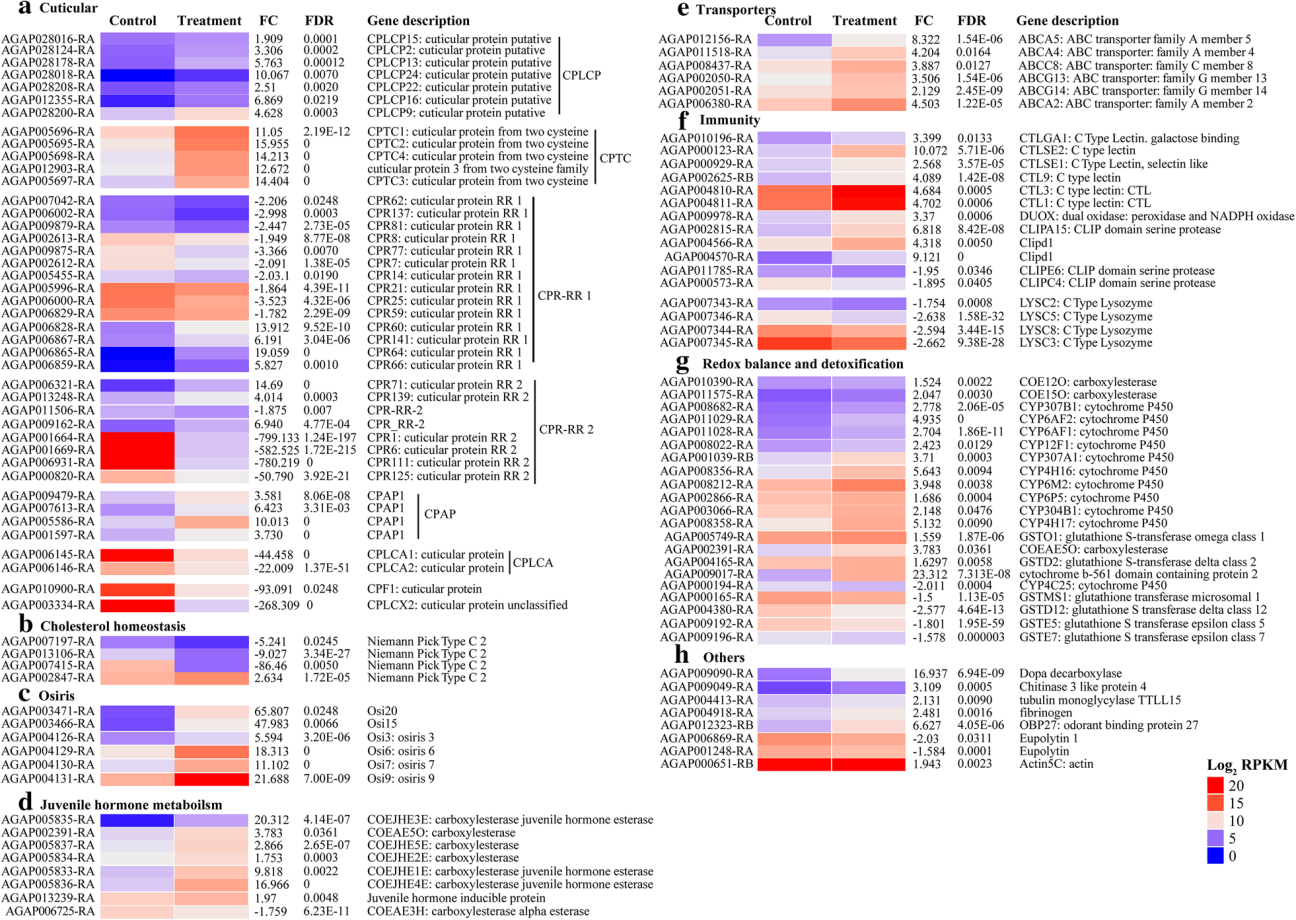
Heat maps representing differentially expressed genes in respective functional categories (**a**–**h**). Heat maps were obtained by plotting the normalized expression profiles (RPKM, log_2_ transformed) of individual genes in control and exposed *An. gambiae* larvae in the R-package software. The heat maps were clustered using Euclidean distance calculation and D. Ward clustering methods. The clusters were then manually separated to various functional categories

About 4.66% (730) of the transcripts were differentially expressed between the control and exposed libraries, most of which (65.75%) were induced by the extract in the exposed libraries (Fig. 3b). The differentially expressed transcripts were predominantly associated with CPs (5.75%), cholesterol homeostasis (0.55%), osiris (0.82%), juvenile hormone metabolism (1.10%), transporters (0.82%), immunity (2.20%), redox balance and detoxification (2.88%) associated gene families among others (1.10%) (Fig. 4). Most (52.4%) of the CPs belonged to the CPR family, within which expression of the RR-2 subfamily was suppressed by the exposure to the extract (Fig. 4a). Expressions of CPLC and CPF families were similarly suppressed and those of CPAP1 induced by the exposure to the extract (Fig. 4a). The exposure also suppressed expression of four Niemann-Pick type C-2 transcripts involved in cholesterol homeostasis (Fig. 4b) while inducing expression of 6 osiris (Fig. 4c), 7 carboxylesterase juvenile hormone esterase (COEJHE) (Fig. 4d), ATP-binding cassette (ABC) transporters (Fig. 4e), 10 immune-related (Fig. 4f) and 11 detoxification associated transcripts (Fig. 4g). The extract exposure suppressed expression of eight C-type lysozymes (Fig. 4f) and four glutathione S transferases (Fig. 4g). Fatty acid elongation and chitin-binding networks were induced while putrescine and ornithine metabolism, ornithine decarboxylase activity, cell wall remodeling, cuticular structural constituents and hydrolytic activities were suppressed by the exposure as revealed by GO analyses (Table 1).

**Table 1:** Gene ontology enrichment analysis of differentially expressed transcripts in exposed relative to control larvae libraries

	GO category	Gene ontology ID	Description of pathway	Test (Ref)	*p*-value
Induced	Biological processes	GO:0006508	Proteolysis	58 (459)	0.0003
		GO:0006030	Chitin metabolic process	11 (210)	0.0025
		GO:1901071	Glucosamine-containing compound metabolic process	11 (210)	0.0034
		GO:0006040	Amino sugar metabolic process	11 (210)	0.0040
		GO:0019367	Fatty acid elongation, saturated fatty acid	2 (4)	0.0074
		GO:0019368	Fatty acid elongation, unsaturated fatty acid	2 (4)	0.0074
		GO:0034625	Fatty acid elongation, monounsaturated fatty acid	2 (4)	0.0074
		GO:0034626	Fatty acid elongation, polyunsaturated fatty acid	2 (4)	0.0074
		GO:0006022	Aminoglycan metabolic process	11 (210)	0.0090
		GO:0030497	Fatty acid elongation	2 (4)	0.0090
		GO:0000038	Very long-chain fatty acid metabolic process	2 (4)	0.0099
		GO:0042761	Very long-chain fatty acid biosynthetic process	2 (4)	0.0099
	Cellular component	GO:0005576	Extracellular region	41 (210)	0.0000
		GO:0044421	Extracellular region part	23 (185)	0.0000
		GO:0005615	Extracellular space	28 (363)	0.0000
	Molecular function	GO:0016787	Hydrolase activity	96 (457)	0.0000
		GO:0004175	Endopeptidase activity	36 (404)	0.0000
		GO:0070011	Peptidase activity, acting on l-amino acid peptides	42 (404)	0.0001
		GO:0008233	Peptidase activity	42 (404)	0.0002
		GO:0008061	Chitin binding	12 (228)	0.0006
		GO:0003824	Catalytic activity	163 (457)	0.0017
		GO:0008236	Serine-type peptidase activity	21 (261)	0.0025
		GO:0017171	Serine hydrolase activity	21 (261)	0.0025
		GO:0004252	Serine-type endopeptidase activity	20 (261)	0.0030
		GO:0102338	3-oxo-lignoceronyl-coa synthase activity	2 (4)	0.0036
		GO:0102337	3-oxo-cerotoyl-coa synthase activity	2 (4)	0.0036
		GO:0102756	Very-long-chain 3-ketoacyl-coa synthase activity	2 (4)	0.0036
		GO:0102336	3-oxo-arachidoyl-coa synthase activity	2 (4)	0.0036
		GO:0009922	Fatty acid elongase activity	2 (4)	0.0036
		GO:0052689	Carboxylic ester hydrolase activity	10 (257)	0.0046
		GO:0004312	Fatty acid synthase activity	2 (4)	0.0056
	KEGG^c^	KEGG:04142	Lysosome	10 (435)	0.0027
Suppressed	Biological Processes	GO:0016998	Cell wall macromolecule catabolic process	4 (132)	0.0005
		GO:0044036	Cell wall macromolecule metabolic process	4 (132)	0.0008
		GO:0071554	Cell wall organization or biogenesis	4 (132)	0.0019
		GO:0009445	Putrescine metabolic process	2 (13)	0.0041
		GO:0009446	Putrescine biosynthetic process	2 (13)	0.0041
		GO:0033387	Putrescine biosynthetic process from ornithine	2 (13)	0.0041
		GO:0006591	Ornithine metabolic process	2 (13)	0.0061
		GO:0050830	Defense response to gram-positive bacterium	3 (38)	0.0090
	Molecular function	GO:0042302	Structural constituent of cuticle	11 (124)	0.0001
		GO:0003796	Lysozyme activity	4 (132)	0.0003
		GO:0004586	Ornithine decarboxylase activity	2 (13)	0.0018
		GO:0004553	Hydrolase activity, hydrolyzing o-glycosyl compounds	6 (80)	0.0031
		GO:0008236	Serine-type peptidase activity	13 (116)	0.0032
		GO:0017171	Serine hydrolase activity	13 (116)	0.0032
		GO:0070011	Peptidase activity, acting on l-amino acid peptides	16 (103)	0.0032
		GO:0061783	Peptidoglycan muralytic activity	3 (38)	0.0033
		GO:0008233	Peptidase activity	16 (103)	0.0039
		GO:0016798	Hydrolase activity, acting on glycosyl bonds	6 (80)	0.0074
		GO:0004252	Serine-type endopeptidase activity	12 (116)	0.0075
		GO:0003824	Catalytic activity	82 (210)	0.0082
		GO:0016787	Hydrolase activity	24 (84)	0.0090

Significantly enriched pathways determined through gProfiler [32]

Test: genes in the differentially expressed dataset; Ref:Entire *Anopheles* genes in gProfiler database; ^C^: Kyoto Encyclopedia of Genes and Genomes (KEGG)

## Discussion

In this study, we used high-throughput RNA-seq expression analysis to determine transcriptional responses in third-fourth instar normal control and abnormal exposed larvae population exposed to sub-lethal concentrations of curry tree (*M. koenigii*) leaf extracts. The extract previously induced morphogenetic abnormalities, reduced locomotion and delayed pupation in the larvae and was predominantly composed of alkaloid phytochemicals (neplanocin A, 3-(1-naphthyl)-l-alanine, lumiflavine, terezine C, agelaspongin and murrayazolinol compounds) [11]. We did not age-match control and exposed populations because of the extract-induced delay in pupation of the exposed relative to control larvae coupled to our need to compare L_3_/L_4_ populations between the treatments (exposed *vs* control). Thus, our molecular results would reflect responses to the inevitable combination of age differences between the larvae populations and the xenobiotic (extract) challenge. We hereinafter refer to this combination as ‘exposed’ and the unexposed normal control as ‘control’ treatments.

We initiated our study by establishing baseline transcriptional processes (molecular investments) that underpin the larval developmental stage in the mosquito control (in the absence of xenobiotics). Our evidence suggests a significant investment in the development and reorganization of musculature in the larvae as evidenced by preferential expression of muscle and cytoskeleton related transcripts [45] in our study. We observed potentially enhanced investment in larval molting and pupation as shown by predominant expression of hexamerins in the larvae. The hexamerins are conserved hemolymph proteins secreted by larval fat body [46] and facilitate larvae-pupae transition [47]. Hexamerins facilitate these processes through regulation of protein reserves (for amino acids and energy) [48], transport of ecdysteroids [49] and juvenile hormone (JH) [50]. We were thus interested in understanding how these and related processes were affected by the extract exposure treatment we have described above in relation to the associated phenotypes we observed in the larvae [11].

Our analysis of processes affected by exposure to the extract revealed the impairment of exoskeleton development, immunity, detoxification processes and transport system by the exposure. Most affected processes were associated with the cuticle that typically protects insects against adverse environmental conditions and pathogens [36]. In that respect, the extract appeared to soften the cuticle through reduced transcription of the RR-2 subfamily of the CPR cuticle family [40, 51] that encodes hard cuticle proteins of the insect exoskeleton [52] and CPLC genes implicated in increasing the thickness of the cuticle through forming rigid matrices [53]. The reduction in hard cuticle synthesis could in turn enhance susceptibility of the larvae to insecticides [54], including pyrethroids [55]. The reduced synthesis is also a possible precursor to the post-ecdysial molting deformities [56] we previously observed in this mosquito [11]. The suppression of the RR-1 subfamily, CPLCPs, CPFs and Niemann-Pick Type C-2 transcripts, and putrescine, ornithine and ornithine decarboxylase pathways could cause enhancement of the deformities due to the exposure. Suppression of the RR-1 subfamily, CPLCPs and CPFs has been shown to potentially interrupt endocuticle development and impede ecdysis, consequently enhancing molting deformities in the mosquito [56, 57]. Niemann-Pick type C-2 controls sterol homeostasis and steroid biosynthesis precursors for ecdysteroids [58]. The ecdysteroids in turn induce larval molting and metamorphosis [59] and promote pupal commitment at L_4_ [60]. The putrescine, ornithine and ornithine decarboxylase pathways are critical for optimal tissue growth and development [61]. Suppression of their expression in our study potentially perturbs cellular processes essential for cytoskeletal structure in the larvae [62]. The interruption of endocuticle development and ecdysis was putatively enhanced by potentially untimely (18 dpe) induction of carboxylesterases juvenile hormone esterase (COEJHE) expression, which could adversely affect pupation [63]. The induction of COEJHE potentially decreased JH hormone titers [64] and initiated a premature pupation process by changing tissue commitment from larval tissue synthesis to production of pupal tissues [65]. This potentially induced the larvae-pupae-transition arrest in our previous observation [11]. The extract appears to target cuticle metabolism in regulating the growth of the larvae.

The larvae putatively counteracted the exposure treatment through induction of osiris, ATP-binding cassette (ABC) transporters, cytochrome P450s (CYPs) and carboxylesterases transcripts and pathways associated with fatty acid (FA) elongation and chitin binding. These transcripts and pathways potentially facilitated interim survival of the larvae in the xenobiotic (extract) permeated aquatic environment. Osiris facilitates the phenotypic plasticity and toxicity responses essential in development, toxicology defenses and digestion in insects [66]. The ATP-binding cassette (ABC) transporters and CYPs facilitate transformation and elimination of endogenous and exogenous compounds including insecticides [67] and phytochemicals [68] by insects. The fatty acid (FA) elongation and chitin-binding pathways trigger intrinsic adaptation mechanisms in insects [55, 69]. However, the detoxification process appeared to be potentially counteracted by suppression of glutathione S transferase (GST) expression by the extract. This group of supressed GSTs is associated with the reduction of oxidative stress in mosquitoes since not all GSTs are involved in reducing oxidative stress [70]. The reduction in GSTs could have also exposed the larvae to oxidative stress [70], suppressed their immunity to bacterial infection [71] and hence enhanced their susceptibility to exogenous phytochemicals [72]. The immunity appears to have been further depressed by suppressed expression of C-type lysozyme in the larvae by the exposure. Lysozymes degrade macromolecules including toxic phytochemicals and have a potential role in innate immunity [73]. The exposure induced the C-type lectin (CTL) immune transcripts [74] in the larvae. The underlying physiological processes underpinning this (CTL) immune response remain to be determined. We similarly observed induction of CPAP, whose role has not been elucidated in *An. gambiae* [75]. Survival in the larvae in the presence of the extract seems to be dependent on opposing forces between those counteracting and those facilitating susceptibility of the larvae to xenobiotics, which include our extract phytochemicals. Overall, our studies reveal significant modulation of several transcripts, some of which may not be directly related to our treatments, but are inherently perturbed, irrespective of external stimuli in the mosquito. These transcripts can potentially be identified from the rest of the differentially expressed transcripts through carefully planned and executed functional genomics studies.

These results have potential implications for integrated vector management (IVM) of *An. gambiae *s.s mosquitoes. First, the exposure treatment appears to impair the cuticular integrity in the larvae, which enhances susceptibility of the larvae to insecticides. This suggests that the exposure treatment can augment the efficacy of insecticide formulations to surmount resistance to insecticides in the mosquito. The current studies were limited to the L_3_/L_4_ instar larval stages of the mosquito, necessitating further studies to establish the efficacy of the extract against younger larvae (below L_3_ instar) and pupae but comparing their underlying molecular processes. Second, the larvae appear to counteract the effect of exposure by instigating adaptive mechanisms, which can be further investigated through generational laboratory or natural chronic exposure experiments and assessment of the resultant biological cost of the adaptation to fitness in the mosquito. The findings will provide additional insight into the impact of such adaptation on the vectorial capacity of the mosquito following temporal exposures of the larvae. The exposure appears to perturb JH metabolism probably because of the Neplanocin A constituent of the extract [11]. Neplanocin A has a similar inhibition effect on JH action as 3-deazaneplanocin A in mosquitoes [11, 76], suggesting potential application of Neplanocin A as a potent JH antagonists. However, these putative responses present challenges and are confounded by age (exposed larvae were older) and physiological compromise (abnormal) in the larvae.

## Conclusions

*Anopheles gambiae *s.s larvae heavily invest in cuticular development, which was disrupted by the exposure treatment. This disruption (exposure) potentially induced the gross morphogenic abnormalities that we previously observed in the larvae exposed to the extract [11]. The cuticle genes can potentially be targeted in the development of more efficacious curry tree phytochemical-based IGRs against *An. gambiae *s.s mosquito larvae. Survival of the larvae in the presence of the extract appears to be dependent on their ability to withstand oxidative stress associated processes induced by the extract. Further studies may adapt these findings for IVM against *An. gambiae *s.s*.* However, such studies should be preceded by validation evaluations that will effectively break down the individual effects of the extract exposure from the confounding factors of age and larvae abnormality we have alluded to above in our results.

## Supplementary Information

**Additional file 1: Table S1.** Primers utilized for *An. gambiae* larvae RNA-transcriptome validation.

**Additional file 2: Table S2.** Analysis of *An. gambiae* larvae RNA-seq libraries. **Sheet 1.** RNAseq dataset output of *An. gambiae* larvae transcripts. **Sheet 2**. Differentially Expressed genes (FC ≥ 1.5) **Sheet 3.** The top 200 most abundant transcripts (RPKM) in the control *An. gambiae* larvae differentially expressed library.

**Additional file 3: Text S1.** Validation of *An. gambiae* RNA-seq results with qPCR.

## Data Availability

The raw transcriptomes datasets generated and/or analyzed during the current study are available in the Sequence Read Archive (SRA) of the National Center for Biotechnology Information (NCBI), USA, under the study Accession number PRJNA560504.
