# Supplementary material for: Transcriptomic response of Anopheles gambiae sensu stricto mosquito larvae to Curry tree (Murraya koenigii) phytochemicals
Source: Parasit Vectors. 2021 Jan 2;14:1. doi: 10.1186/s13071-020-04505-4 (PMC7777392; doi:10.1186/s13071-020-04505-4)
Supplement: Supplementary file 1 — Additional file 1: Table S1. Primers utilized for An. gambiae larvae RNA-transcriptome validation. [file 13071_2020_4505_MOESM1_ESM.pdf]

Primer list used for *Anopheles gambiae s.s* larvae transcriptome validation

**VectorBase Gene ID**	**VectorBase Description**	**Forward Primer**	**Reverse primer**	**Size (bp)**	**Annealing Temp (ºC)**
AGAP006000-RA	CPR25: cuticular protein RR-1 family 25	TGTGCATTGATCTGTGTGGC	GATGCCGTTGCTAGTCTCGT	144	56.8
AGAP010617-RA	Unknown	CACTTGCATCAGCCGGAGTA	GCACAAGTAGCCTCCGATGT	136	56.8
AGAP011277-RA	Unknown	GATGGAGAGTTCTGGCTCGG	ACAGCTTCACCTCACTGTCG	148	63.2
AGAP002810-RA	45 kDa calcium	AAGCCGGAAGTGGTGTATCG	CTTGTCGCCATTTGTGTCGG	131	63.2
AGAP003471-RA	Osi20	ACACCAAAGCGAAAACGCAA	TTGGACGGTACCACTGTTCG	148	55.9
AGAP005833-RA	COEJHE1E: caboxyleste se juvenile hormone esterase	GACCACTTCGTTTCGCCAAC	CCTCCTTACCCTCGACTCCA	141	63.2
AGAP008781-RA	Elongation of very long chain fatty acids protein 5	TATGTCGATGTCCTGCGGTG	TTCTGTTTCTTGCGCAGCAC	144	55.0
AGAP009017-RA	Cytochrome b-561 domain containing protein 2	GAACCGGTACTCGACTGCAA	CCCGTTATGGCATGATCCGA	150	63.2
AGAP009623-RA#	GAPDH	GTTCATCGGCGTCGACTACA	CTCCTGGAACACGGCAATCT	130	56.8
AGAP010362-RA#	Unknown	CAACGGGGCTTCAGACGATA	TACCACAAGTGATGCCACGG	147	55.0
AGAP011790-RB#	CLIPA2: CLIP-domain serine protease	TCACCCAGTGCGACAGTAAC	GAAACTCCTCCGGAAAGGCA	138	55.0
AGAP028065-RA#	Unknown	TGTACGATCATGGCAGCGAA	GAAGCCACTTGACCTTTGCG	144	62.0
