# Supplementary material for: Transcriptomic response of Anopheles gambiae sensu stricto mosquito larvae to Curry tree (Murraya koenigii) phytochemicals
Source: Parasit Vectors. 2021 Jan 2;14:1. doi: 10.1186/s13071-020-04505-4 (PMC7777392; doi:10.1186/s13071-020-04505-4)

Validation of mosquito larvae RNA-seq results with qPCR. The expression values (log2 ratios) for eight genes are plotted against qPCR values (log2 ratios). The Pearson correlation coefficient **(R=0.978**) and Goodness fit, **R^2^ = 0.956** obtained indicate high correlation. These results indicate that the qPCR correctly validates mosquito larvae RNA-seq data.

| **VectorBase Gene ID** | **Protein Name** | **Fold change in qPCR** | **Log2 of qPCR** | **Fold change in RNA-seq** | **Log2 of RNA-seq** |
| --- | --- | --- | --- | --- | --- |
| AGAP006000-RA | CPR25: cuticular protein RR-1 family 25 | -4.182269 | -2.064000 | -3.523449 | -1.816989 |
| AGAP010617-RA | Unknown | -19.678815 | -4.299000 | -18.440174 | -4.204780 |
| AGAP011277-RA | Unknown | -6.753869 | -2.756000 | -16.975791 | -4.085407 |
| AGAP002810-RA | 45 kDa calcium | 3.450111 | 0.929000 | 2.298492 | 1.200688 |
| AGAP003471-RA | Osi20 | 6.282891 | 2.651000 | 65.807495 | 6.040180 |
| AGAP005833-RA | COEJHE1E: caboxylesterase juvenile hormone esterase | 2.259047 | 1.176000 | 9.817610 | 3.295372 |
| AGAP008781-RA | Elongation of very long chain fatty acids protein 5 | 6.032923 | 2.593000 | 32.902245 | 5.040114 |
| AGAP009017-RA | Cytochrome b-561 domain containing protein 2 | 5.138790 | 2.361000 | 23.312790 | 4.543050 |


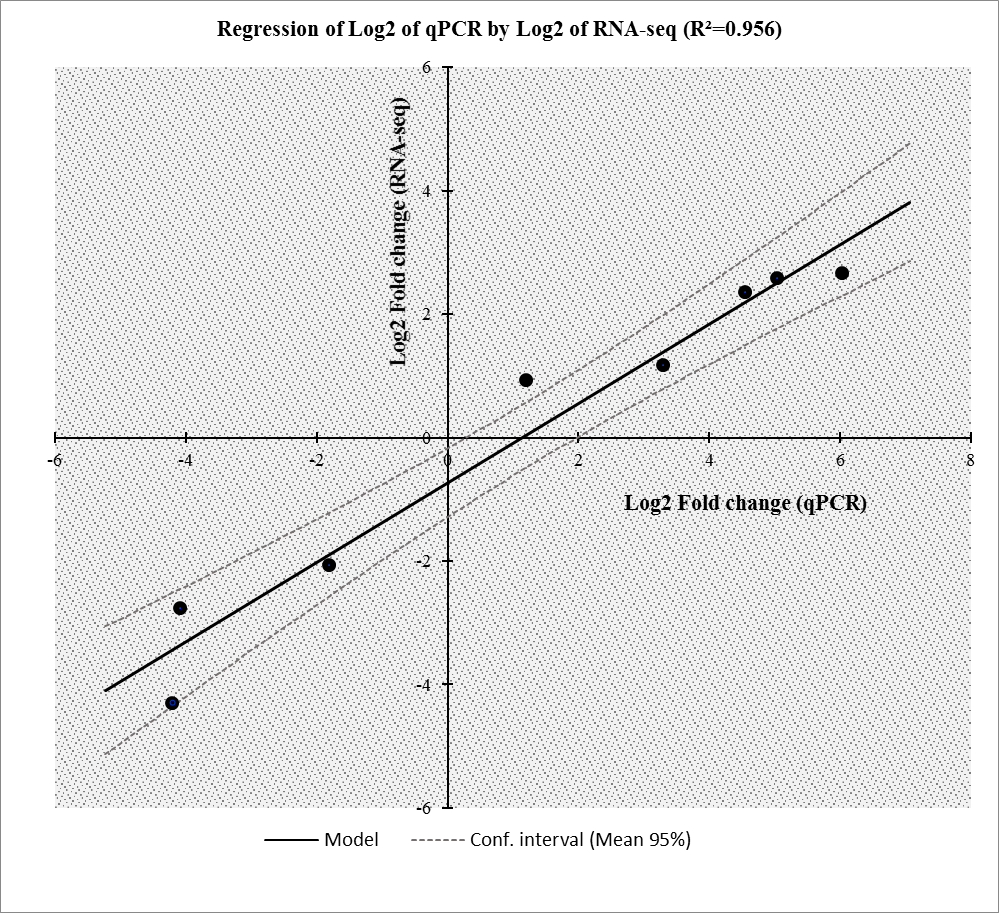

Supplement: Supplementary file 3 — Additional file 3: Text S1. Validation of An. gambiae RNA-seq results with qPCR. [file 13071_2020_4505_MOESM3_ESM.docx]
